# Neutrophil dysregulation during sepsis: an overview and update

**DOI:** 10.1111/jcmm.13112

**Published:** 2017-02-28

**Authors:** Xiao‐Fei Shen, Ke Cao, Jin‐peng Jiang, Wen‐Xian Guan, Jun‐Feng Du

**Affiliations:** ^1^ Department of General Surgery Affiliated Drum Tower Hospital of Nanjing University Medical School Nanjing China; ^2^ Department of Intensive Care Unit Affiliated Drum Tower Hospital of Nanjing University Medical School Nanjing China; ^3^ Department of Rehabilitation Medicine PLA Army General Hospital Beijing China; ^4^ Department of General Surgery PLA Army General Hospital Beijing China

**Keywords:** sepsis, neutrophil migration, neutrophil antimicrobial activity, neutrophil extracellular traps, signal pathway

## Abstract

Sepsis remains a leading cause of death worldwide, despite advances in critical care, and understanding of the pathophysiology and treatment strategies. No specific therapy or drugs are available for sepsis. Neutrophils play a critical role in controlling infection under normal conditions, and it is suggested that their migration and antimicrobial activity are impaired during sepsis which contribute to the dysregulation of immune responses. Recent studies further demonstrated that interruption or reversal of the impaired migration and antimicrobial function of neutrophils improves the outcome of sepsis in animal models. In this review, we provide an overview of the associated mediators and signal pathways involved which govern the survival, migration and antimicrobial function of neutrophils in sepsis, and discuss the potential of neutrophils as a target to specifically diagnose and/or predict the outcome of sepsis.

## Introduction

Sepsis is very common and remains a major cause of death in hospitals. Possibly due to the progressive ageing of population and the increasing recognition of the disease, as well as more frequent and liberate use of sepsis codes after hospital discharge, the incidence of sepsis has increased over the past years 1. The prevalence of sepsis is estimated to be 300 cases per 100,000 worldwide 2. Large‐scale epidemiological study of the incidence and mortality rate of sepsis in China is rare, and it is estimated that the incidence of sepsis is 461.0 cases per 100,000 with a mortality rate of 79.0 deaths per 100,000. Although studies from different countries around the world have demonstrated a decrease in mortality rate of sepsis over the past years 3, 4, 5, the absolute number of patients who die of sepsis is increasing on account of the increased incidence 1. As a most recent study has suggested that the current definition of severe sepsis used currently might miss one in eight patients who were actually with infection, organ failure and substantial mortality 6, the incidence and mortality rate of sepsis worldwide may be underestimated.

Numerous efforts have been made to improve the outcome of sepsis, including the use of standards for the diagnosis of sepsis and supportive therapies according to the Surviving Sepsis Campaign 7, the administration of antibiotics when indicated, and surgical or interventional radiological approaches for eliminating and/or controlling the source of infection 8. However, sepsis is still the leading cause of morbidity and mortality in hospitalized patients 9, and patients with sepsis also experienced a longer length of stay with eight times more likely to die during hospitalization than other inpatients 10, 11. Currently, no specific drugs for sepsis are available, and drotrecogin alfa (recombinant human activated protein C), the only approved drug for severe sepsis treatment, was also withdrawn because it did not significantly reduce mortality in patients with severe sepsis and septic shock in several multicenter clinical trials 12, 13.

Neutrophil are the first line of defence in protecting the body from infection. Sepsis is defined as uncontrolled host responses induced by the microbial invasion 8 and failure of neutrophils in the infection sites with dysregulated immune responses to infection has been largely reported previously 11, 14, 15, 16. Thus, re‐evaluating of the pathophysiology of how sepsis influences the function and migration of neutrophils, as well as the signal pathways involved, may help to develop new and promising treatment strategies for sepsis. Moreover, based on the dysregulated characteristics of neutrophils in sepsis, potential targets on neutrophils as diagnostic and/or prognostic biomarkers for sepsis are also discussed.

## Recent advances in the pathophysiology process of sepsis

Sepsis has now been defined as life‐threatening organ dysfunction caused by a dysregulated host response to infection 17, 18 and is a complex interplay of host pro‐inflammatory and anti‐inflammatory processes that determines the fate of the individuals. Traditionally, the pathophysiology of sepsis was considered to be an initial hyperinflammatory phase which lasts several days and subsequently a more protracted immunosuppressive phase 9. The previous definition of sepsis based on systemic inflammatory response syndrome (SIRS) criteria focused solely on inflammatory excess 19. However, recent studies have shown that both pro‐inflammatory and anti‐inflammatory responses occur early with hyperinflammatory response much more predominant 20, 21, along with major modifications in non‐immunologic pathways such as cardiovascular, neuronal, metabolic and coagulation 18. Moreover, in contrast to infection plus an accompanying inflammatory response alone, sepsis often involves organ dysfunction 18. With improved therapeutic strategies in humans, most patients have survived the hyperinflammatory phase 22, but failed to achieve significantly improved outcome due to the failure of the primary infection control or the acquisition of secondary hospital‐acquired infections 23. Direct evidence of the pathophysiology of sepsis comes from a study in which investigators acquired the spleen or lungs of patients with sepsis within 30–180 min. of death and found that these patients had marked immunosuppression 24. Both innate and adaptive immune systems were involved in this immunosuppression process, with a marked decreased in the production of pro‐inflammatory and anti‐inflammatory cytokines in immune cells. The inhibitory factors including the increased expression of PD‐1, expansion of regulatory T cells and myeloid‐derived suppressor cell populations, and down‐regulation of CD28 and HLA‐DR‐mediated activation pathways were also discovered in this study 24. These results were consistent with findings from other studies which showed that pro‐inflammatory cytokine production in peripheral blood mononuclear cells and whole blood from patients with sepsis was significantly decreased with anti‐inflammatory cytokine production increased 25, 26. Studies have also suggested that a large number of patients who died of sepsis had unresolved opportunistic infections 27, 28 and paediatric patients who died due to sepsis also showed similar immunosuppressive features to those observed in adult patients with sepsis 9, 29; thus, immunosuppression may be the predominant driving force with regard to morbidity and mortality in sepsis.

Immune cell apoptosis, which has been confirmed to occur in all ages of groups (neonatal 30, paediatric 31 and adult population 24), is thought to contribute a lot to the development of the immunosuppressive pathophysiology of sepsis. In the innate immune system, most innate cells tend to undergo apoptosis, such as dendritic cells, immature macrophages and natural killer cells, whereas neutrophils displayed delayed apoptosis. However, the function of neutrophils is impaired 32, and deleterious accumulation within remote vital organs is also directly correlated with the increase in sepsis‐induced morbidity and mortality 23. In the adaptive immune system, CD4 + and CD8 + T cells, as well as B cells, show a marked loss, whereas regulatory T cells display resistance to sepsis‐induced cell apoptosis. Immune cell apoptosis not only leads to defects in the fight against the remaining pathogenic organisms and opportunistic pathogens, but also results in immune tolerance which is mainly induced by the surviving immune cells taking up apoptotic cells. Therapies that prevent apoptosis such as anti‐apoptotic cytokines, caspase inhibitors and death receptor antagonists which prevents lymphocyte apoptosis have been shown to improve survival in sepsis 33, 34, 35. As neutrophils are crucial components during sepsis and show delayed apoptosis with impaired function and trafficking, we will further discuss how these might happen in the following sections.

## Effects of sepsis on neutrophils

### Increased lifespan of neutrophils during sepsis

**Figure 1:**
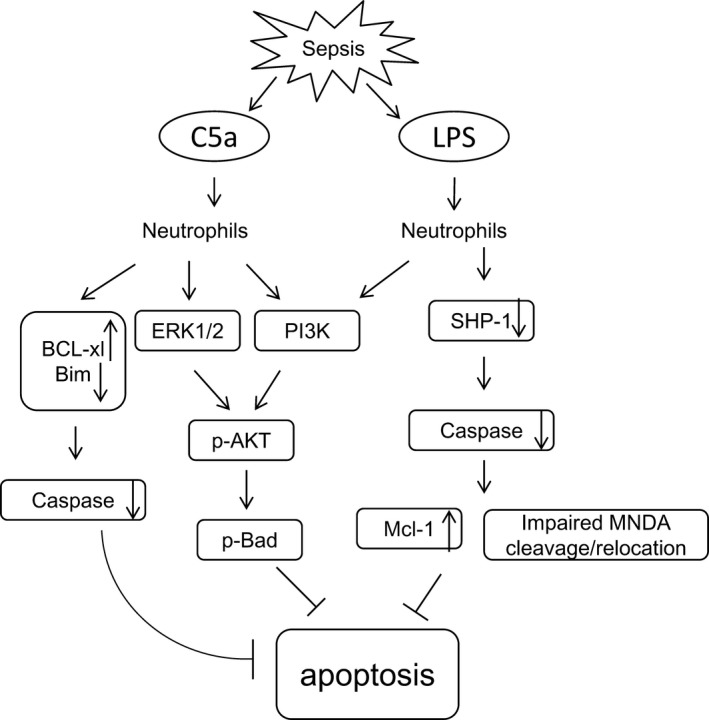
Schematic depicting signal pathways of impaired neutrophil apoptosis during sepsis. During sepsis, C5a and LPS mainly mediate the increased lifespan of neutrophils through multisignal pathways. The activation of ERK1/2 and PI3K pathways by C5a and/or LPS can lead to the phosphorylation of Akt and subsequent phosphorylation of Bad, which inhibits cytochrome C release from mitochondria to prevent the formation of apoptosome. C5a can also enhance Bcl‐xL expression and reduce Bim expression. Moreover, LPS negatively influences MNDA relocation and cleavage and prevents proteasomal degradation of MCL‐1. All of these events favour the prolonged survival of neutrophils during sepsis.

Under normal conditions, circulating neutrophils have a short half‐life (7–12 hrs *in vivo*), but have an increased lifespan during sepsis. The level of the anti‐apoptotic protein Mcl‐1 (myeloid cell leukaemia) which plays a key role in neutrophil apoptosis was found to be increased in neutrophils isolated from patients with severe sepsis 36. Several signal pathways have also been discovered to contribute to neutrophil resistance to apoptosis. Pro‐inflammatory mediators such as lipopolysaccharide (LPS) and complement component 5a (C5a) in the peripheral circulation can induce the activation of extracellular regulated protein kinases (ERK) 1/2 and phosphoinositide ‐3 kinases (PI‐3K) in neutrophils, which lead to the increased anti‐apoptotic protein Bcl‐xL expression and decreased levels of Bim expression 37, 38. The phosphorylation of Akt can also result in the phosphorylation of Bad, which prevents the formation of the apoptosome and inhibits neutrophils apoptosis 39. In addition, exposure to LPS can also reduce the activity of the Src‐homology domain 2 containing tyrosine phosphatase‐1 (SHP‐1) and its binding to caspase‐8 with the increased expression of non‐receptor tyrosine kinase Lyn in neutrophils 40. The reduced activity of capase‐8 then leads to the impaired cleavage/relocation of nuclear factor MNDA (myeloid nuclear differentiation antigen) in parallel with an accumulation of Mcl‐1 and together results in the suppression of the neutrophil apoptosis 41 (Fig. 1). Other apoptosis pathways such as extrinsic pathway which consists of tumour necrosis factor (TNF)‐α or Fas ligand (FasL) binding to specific receptors on neutrophils have also been suggested to contribute to delayed neutrophil apoptosis in sepsis 42, 43.

### Migration of neutrophils in sepsis

**Figure 2:**
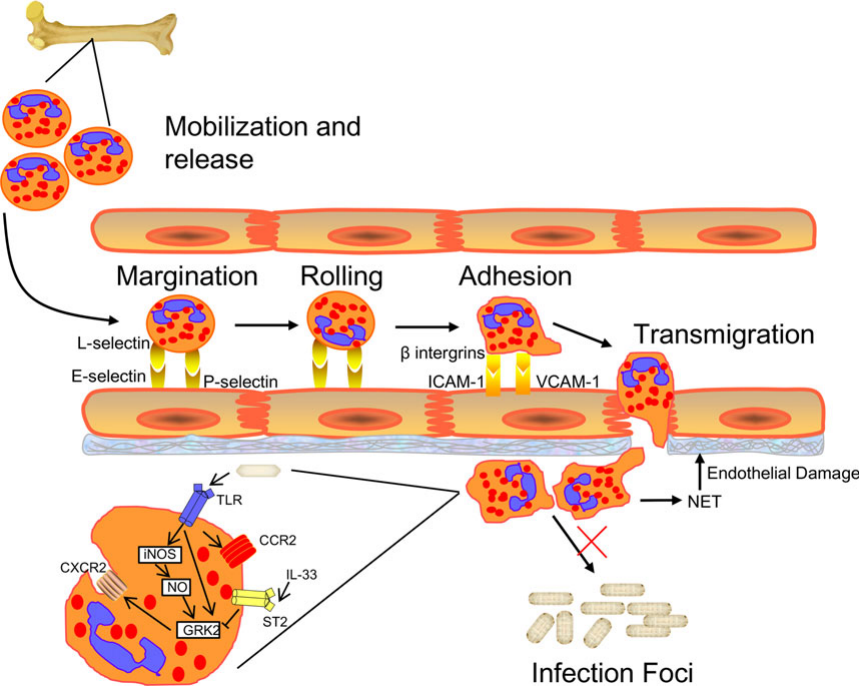
Schematic depicting four phases of neutrophil migration in sepsis and signal pathways which accounts for the impaired migration of neutrophils into infection sites directed by CXCR2. During sepsis, neutrophils are systemically stimulated with impaired migration to the infection foci. Bacterial components can activate TLRs expressed on neutrophils and lead to the up‐regulation of GRK2, resulting in the desensitization of CXCR2 on the surface of neutrophils. Administration of IL‐33 can reverse the effects of GRK2 on CXCR2 expression, driving neutrophils migrating to the site of infection. In addition, activation of TLRs can also up‐regulate CCR2 on the surface of neutrophils, favouring the recruitment of neutrophils to distant organs.

The migration of neutrophil *in vivo* includes four distinct phases, all of which are impaired during sepsis: mobilization and release from the bone marrow, margination and rolling, adherence, and transmigration 15. The mechanisms which contribute to the development of sepsis‐induced impairment of neutrophil migration have also been investigated in numerous studies (Fig. 2).

### Release of neutrophils

In the bone marrow, granulocyte colony‐stimulating factor (G‐CSF) and granulocyte–macrophage colony‐stimulating factor (GM‐CSF) mainly direct the granulopoiesis process 23, whereas chemokines and adhesion molecules expressed on neutrophils and bone marrow endothelia cells play central roles in regulating neutrophil release into the blood. Under normal conditions, the balance of chemokines and their receptors (C‐X‐C chemokine receptor (CXCR)4 interacting with its ligand C‐X‐C chemokine receptor ligand (CXCL)12 to mediate retention and CXCR2 interacting with CXCL1 or CXCL2 to mediate release) help to maintain neutrophils within the bone marrow, with only a small fraction of mature neutrophils released into the blood. During sepsis, pro‐inflammatory cytokines such as tumour necrosis factor (TNF)‐α, interleukin (IL)‐1β, IL‐6 and IL‐17 and bacterial products could up‐regulate the level of G‐CSF which promotes both the generation of both mature and immature neutrophils. In addition, the expression of CXCL12 is down‐regulated in sepsis while CXCL1 increases, which drives the release of neutrophils into the blood 44, 45. Subsequent studies have demonstrated that the CXCR4 and CXCL2 interaction plays a central role in mediating neutrophil release without the requirement of other signalling pathways, such as Toll‐like receptor (TLR)4, myeloid differentiation primary response gene (MyD)88 or TIR domain‐containing adaptor‐inducing interferon‐β (TRIF) 45.

### Alterations in neutrophil rigidity and adhesion

The margination and rolling phase of neutrophil migration requires cellular deformability and the expression of endothelial E‐selectin and P‐selectin, which display low‐affinity interactions with l‐selectin expressed on the surface of neutrophils 46. During sepsis, bacterial products and pro‐inflammatory cytokines such as TNF‐α and IL‐1β promote the shedding of l‐selectin and stimulate the expression of β‐integrins on the cell surface of neutrophils, which interact with intercellular adhesion molecule‐1 (ICAM‐1) and vascular cell adhesion molecule‐1 (VCAM‐1) on the vascular endothelium and promote high‐affinity adhesion with the endothelium 23. The expression of β1‐ and/or β2‐integrins is relatively low in neutrophils under normal conditions and is up‐regulated by different bacterial products 47. As a result, neutrophils display reduced margination and rolling with reduced deformability, and sequester in the vascular compartment. Capillary bed sequestration of neutrophils further leads to vascular occlusion and promotes tissue ischaemia and organ dysfunction, especially in the lung and liver which are rich in blood vessels. *In vitro* results have also substantiated these findings, whereas neutrophil rigidity can be induced by TNF‐α and is mainly mediated by the activation of peroxisome proliferator‐activated receptor gamma (PPARγ) which induces the accumulation of deformability related F‐actin below the cell membrane 48.

### Impairment of neutrophil transmigration

The transmigration of neutrophils involves passage through tight junctions in the endothelium and further into the primary infected foci, which requires leucocyte chemoattractants, such as formyl–methionyl–leucyl–phenylalanine (fMLP), platelet activating factor, C5a, leukotriene B4 and IL‐8, binding with chemokine receptors expressed on neutrophils 14, 23. Evidence from mice and patients with severe sepsis has demonstrated inadequate migration of neutrophils to the infection foci 32, despite the fact that high levels of chemokines at the infection site were induced during severe sepsis 49. The mechanisms accounting for the inability of neutrophils to migrate to infection foci have largely been described. CXCR2 was found to be reduced in the neutrophils of mice and patients with severe sepsis 50, 51, 52, and neutrophils in these patients also displayed decreased chemotaxis response to fMLP, leukotriene B4 or IL‐8 *in vitro*. The mechanisms underlying the down‐regulated expression of CXCR2 have been studied in detail. Prolonged or repeated stimulation by chemokines and ligands could lead to the activation of G‐protein coupled receptors (GPCRs), which recruit GPCR kinases (GRKs) to the plasma membrane of neutrophils. The subsequent GRK phosphorylation of GPCRs on their C‐terminus results in the functional desensitization of the chemokine receptor in a β‐arrestin‐ and clathrin‐dependent manner 53, 54. The activation of TLR 2, 4 and 9 in response to lipoteichoic acid, LPS and CpG‐oligodeoxynucleotide can also induce the internalization of CXCR2 by up‐regulating GRK2 expression during sepsis in mice 55, 56. Nitric oxide (NO), which can be produced by numerous cells under the stimulation of inflammatory cytokines and bacterial products in an inducible nitric oxide synthase (iNOS)‐dependent pathway, is of significant importance in the antimicrobial activity of neutrophils 57, and complete deficiency of this mediator can increase bacterial burden 58. Moreover, enhancement of NO during sepsis can trigger the activation of soluble guanylate cyclase (sGC), cyclic‐GMP formation and protein kinase G (PKG) phosphorylation, all of which induce the expression of GRK2 in neutrophils, and promote the internalization of CXCR2 59 (Fig. 2). The PI3Kγ signalling pathway is thought to be involved in the dimerization of iNOS 60; thus, investigators have studied the role of PI3K in regulating neutrophil migration in sepsis. *PI3Kγ*−/− mice with sepsis displayed increased CXCR2 expression with reduced GRK2 expression and had higher survival rates 61. In addition, *in vitro* results also confirmed that *PI3Kγ*−/− neutrophils incubated with CXCL2 showed decreased internalization of CXCR2 61. Therefore, PI3K also serves as a negative regulator in the process of CXCR2 internalization. Similar to the PI3K pathway in controlling neutrophil migration, intracellular production of PPARγ in neutrophils also serves as a negative regulator of neutrophil migration in a NO‐ and ERK1/2‐dependent manner 62, 63. The lectin‐like oxidized low‐density lipoprotein receptor‐1 (LOX‐1), which recognizes inflammatory products such as C‐reactive protein (CRP), apoptotic cells, bacterial products and activated platelets, has also been suggested to play a role in the internalization of CXCR2 as depletion of this gene prevented the down‐regulation of CXCR2 and improved neutrophil migration during sepsis 64. Despite the effect of cytokines and bacterial products, other mediators such as acute phase proteins 65, the enzyme heme oxygenase (HO)‐1 66, elevated temperatures 67, hemopexin 68 and microparticles released from numerous cell populations during sepsis 69 also play a role in the failure of neutrophil migration into infection sites. In addition to the dysregulated expression of CXCR2 on neutrophils, alterations in other relevant chemokine receptors also occur during sepsis, which promote aberrant neutrophil trafficking into remote organs 70, 71. Studies have suggested that CCR2, which is primarily not expressed on neutrophils under certain circumstances, could be expressed on circulating neutrophils in a TLR‐dependent manner and contributes to the development of random migration of neutrophils 70.

Mediators which functions through neutrophils and protect the body against sepsis have also been described. IL‐33, which binds to the heterodimeric receptor complex ST2, can block TLR‐4 induced internalization of CXCR2 through the inhibition of GRK2 and improves the recruitment of neutrophils to the infection foci 72. The cystathionine b‐synthase and cystathionine g‐lyase (CSE) enzymes, which help to synthesize hydrogen sulphide (H_2_S) from l‐cysteine, have also been suggested to act as a negative feedback regulators in the development of sepsis by regulating neutrophil CXCR2 expression 73.

## Alteration of neutrophil function in sepsis

### Antimicrobial activity of neutrophils in sepsis

The antimicrobial activity of neutrophils includes the recognition of invading pathogens and microbial components, the subsequent signalling pathway leading to the release of inflammatory cytokines and chemokines, oxidant generation, phagocytosis and the formation of neutrophil extracellular traps (NETs) 23, 74, 75. All of these effector functions of neutrophils are thought to be influenced by sepsis. TLRs on neutrophils help to recognize pathogen‐associated molecular patterns (PAMP) or danger‐associated molecular patterns (DAMP) 76. The activation of TLRs promotes the downstream release of pro‐inflammatory cytokines, chemokines and antimicrobial peptides in a nuclear factor (NF)‐κB‐ and mitogen‐activated protein kinase (MAPK)‐dependent manner, as well as the generation of reactive oxygen species (ROS) 77. Appropriate activation of TLRs is beneficial to the body against pathogens, whereas persistent activation has led to the tolerance of the TLRs signal pathway with reduced expression of pro‐inflammatory cytokines and up‐regulation of TLRs signal inhibitors such as NFκBIA both in animal and in human studies 78, 79, 80. The overwhelming bacterial load during sepsis can also activate the complement system, in which the high levels of C5a can significantly diminish migration, phagocytosis and ROS production in neutrophils 81. Microarray analysis also showed that neutrophils acquired from patients with sepsis within 24 hrs of admission showed suppression of several gene clusters, such as inflammatory response genes, immune modulation genes and genes regulating oxidant production 82. Neutrophils from mice subjected to Pseudomonas sepsis demonstrated temporal impairment of oxidant production 83, and the phagocytosis of neutrophils during sepsis was also found to be impaired by mediators expressed by bacteria and cytokines such as IL‐10 84, 85. In human studies, impaired neutrophil function has also been identified. Neutrophils acquired from patients with septic shock displayed a markedly alteration in chemotaxis and oxidative burst capacity, and the number of circulating immature neutrophils also increased 86. These aspects were closely related to an increased risk of death after septic shock although neutrophils in these patients maintained phagocytosis and activation capacity 86. Moreover, neutrophils from septic mice and patients induced lymphocyte apoptosis in a cell‐contact‐dependent manner through the expression of programmed cell death 1 ligand (PD‐L1) and thus promoted the process of immunosuppression during sepsis 87. The level of PD‐L1 on neutrophils was also identified to be positively correlated with the severity of septic patients with an area of 0.74 under the receiver operating curve, which further suggested the potential of neutrophil PD‐L1 as a biomarker for prognosis of patients with sepsis 87.

As previously discussed, NO is involved in the internalization of CXCR2 on neutrophils. In addition, it also regulates the expression of adhesion molecules, reducing leucocyte rolling and adhesion to the endothelium, which results in the failure of neutrophil migration into infection foci. Based on these results, NOS inhibition and/or NO blockage were used and were shown to increase neutrophil recruitment to the peritoneal cavity in caecal ligation and puncture (CLP)‐induced sepsis through enhanced interaction with the endothelium 58. However, the pharmacological inhibition of iNOS for extended periods of time did not allow animals with increased bacterial burden to escape death 58. NO is crucial for neutrophils to kill bacteria and total absence of this mediator may lead to neutrophils being unable to control local infection despite the presence of enhanced migration to infection loci. Thus, therapy involving NOS inhibition and/or NO blockage should be carefully evaluated.

### Neutrophil extracellular traps (NETs) in sepsis

Recently, NETs have been shown to play an important role in neutrophil antimicrobial activity and are composed of a network of chromatin fibres associated with granules of antimicrobial peptides and enzymes such as myeloperoxidase, elastase and cathepsin G, which capture and kill invading microbial pathogens 88. The formation of NETs depends on the exposure of neutrophils to bacterial 89 and neutrophil elastase released from azurophilic granules, which can decondensate the nuclear chromatin and further confer antimicrobial properties along with other serine proteases and NET‐associated myeloperoxidase 90, 91. The role of NETs in the control of invading pathogens spreading in sepsis has not been fully elucidated. Some studies have suggested that NETs are important in reducing bacterial spreading 92, especially in the early phase of infection 93, whereas others also observed that NETs are not indispensable in the control of bacterial load as the lack of the enzyme related to NETs formation and treatment of rhDNAse which could prevent the formation of NETs did not result in increased bacterial loads in animals subjected to sepsis 94, 95. Furthermore, excessive formation of NETs has been discovered during sepsis, which is correlated with the development of organ damage. The interaction between neutrophils and activated platelets 96, as well as activated endothelial cells in sepsis 97, was shown to promote the formation of NETs, which could adhere and activate the vascular endothelium, and ultimately lead to endothelial cell damage and organ injury in a histone‐ and myeloperoxidase‐dependent manner 96. In addition, histones can also affect TLR2 and TLR4 to stimulate the production of pro‐inflammatory cytokines through MyD88 signalling 98, 99, 100. As no significant difference was identified in survival rate between rhDNAse‐treated and non‐treated mice subjected to CLP 101, it was proposed that the formation of NETs actually had a deleterious role in sepsis. Nevertheless, the combination of antibiotic therapy and treatment to prevent their formation such as rhDNAse treatment or inhibition of the enzyme peptidylarginine deiminase was shown to improve the survival rate of animals with sepsis 95, 101. Similar results were also obtained in human studies, in which the severity of acute respiratory distress syndrome in patients with sepsis was relative to the level of proteolytic enzymes in the bronchoalveolar 102.

**Figure 3:**
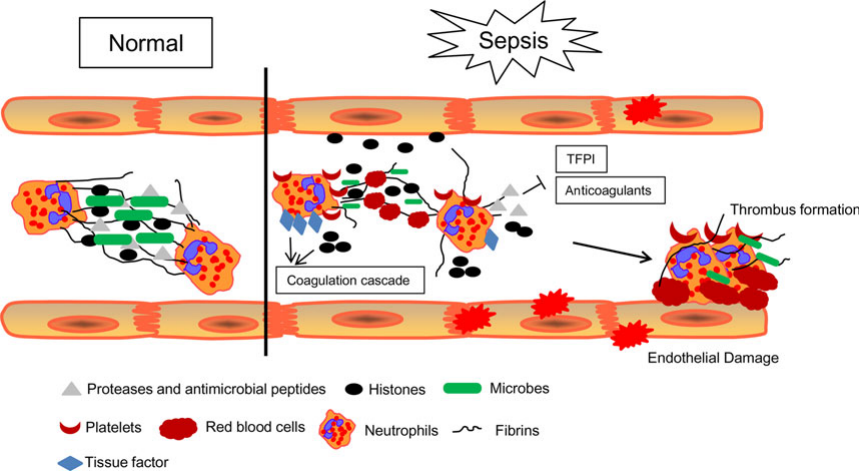
NETs under normal condition and sepsis. The formation of NETs induced by pathogens is crucial for extracellular microbial trapping and killing. During sepsis, neutrophils accumulate and adhere to vascular endothelium in collaboration with platelets. They express tissue factor and expel tissue factor‐bearing NETs which could initiate the coagulation cascade. Moreover, the components of NETs also promote blood coagulation through numerous pathways.

Despite the antimicrobial activity and tissue damage process during sepsis, NETs have also been suggested to contribute to coagulation disturbance in sepsis in recent studies 103, 104. NETs were found to stimulate fibrin formation and deposition *in vitro*, and fibrin can colocalizes with NETs in blood clots 104, 105, 106. Consistent with these results, a recent study has also suggested that NETs provide a platform for platelet and red blood cell adhesion, representing another thrombus scaffold for thrombi, and promote fibrin deposition 107. In addition, tissue factor‐bearing NETs were also found in patients with sepsis 108. Further results showed that NETs could support the activation of factor XII (FXII) which binds to neutrophils, stimulate fibrin formation via the intrinsic coagulation pathway and promote the formation of thrombosis 104. The components of NETs, such as DNA, histones and granule proteins, have all been discovered to provide pro‐coagulant activities 109. DNA can initiate the intrinsic coagulation cascade and nucleic acids can enhance the activity of coagulation serine proteases 110. Histones, which are the most abundant proteins in NETs, could generate thrombin during sepsis through inhibiting anticoagulants and trigger platelet activation, leading to prothrombotic and pro‐coagulant responses 111. In addition, extracellular histones are also cytotoxic to endothelia and epithelial cells 99. Furthermore, neutrophil‐derived granule proteins, especially neutrophil elastase, contribute to thrombus formation by inhibiting tissue factor pathway inhibitor (TFPI) and anticoagulants such as antithrombin (AT) and activated protein C (APC) 106 (Fig. 3).

### Neutrophils as biomarkers for sepsis

The diagnosis of sepsis has always been a central problem as early and accurate diagnosis can lead to appropriate therapy and improved outcome 112. Delayed diagnosis of even a few hours has been shown to result in increased mortality 113. The detection of pathogens in blood cultures is used as the gold standard for the diagnosis of sepsis 114. However, the long‐time taken to obtain culture results and high negative cultures in patients with severe sepsis has largely limited its clinical utility 115.

As mentioned above, neutrophils are involved in the process of sepsis, and a relative increase in the total number of circulating neutrophils or an increase in the percentage of immature forms is also closely related to sepsis. Furthermore, the production of neutrophils, such as toxic granulations and Dohle bodies, are regarded as specific markers of bacterial infection 112. Therefore, activation markers in neutrophils may be potential biomarkers for the diagnosis and/or prognosis of sepsis. Several biomarkers have been investigated for their potential capacity as diagnostic and/or prognostic biomarkers. CD64 expressed on neutrophils and monocytes is the high‐affinity immunoglobulin Fcγ receptor I and mediates the phagocytosis of bacteria. The expression of CD64 on neutrophils is relatively low under normal conditions, whereas a sharp increase in expression up to 10‐fold can be found when neutrophils are activated by pro‐inflammatory cytokines 116, 117. Further studies showed that the percentage of CD64‐positive neutrophils in patients with bacterial infection was significantly higher than in patients with viral infection 118, and the specificity for CD64 expression on neutrophils during bacterial infection also reached 85% and 91% in two systematic meta‐analyses 119, 120. Therefore, CD64 expression on neutrophils is specific for bacterial infection. As sepsis is defined as uncontrolled host response to infection, the use of CD64 expression as a diagnostic biomarker for sepsis induced by bacterial infection has been studied extensively. Results showed that CD64 expression on neutrophils can be a useful diagnostic biomarker for both adult and neonatal sepsis 121, 122, especially in combination with other biomarkers 123. Moreover, CD64 expression could serve as a potential early prognostic marker for sepsis as high levels of CD64 expression are closely related to both the severity and 28‐day mortality of sepsis 124, 125.

The triggering receptor expressed on myeloid cells‐1 (TREM‐1), which is a member of the immunoglobulin superfamily, is expressed on neutrophils, monocytes and macrophages after exposure to LPS, heat‐inactivated bacteria or fungi 126, 127. TREM‐1 has two forms: the membrane form (mbTREM‐1), which is associated with adaptor DAP12 and synergizes with TLRs to amplify inflammatory signals, and the soluble form (sTREM‐1) which acts as a counter‐regulatory molecules to attenuate inflammation. It has been suggested that after challenged by bacteria, the up‐regulation of mbTREM‐1 on neutrophils facilitates their migration across the epithelial barrier and amplifies local inflammation, whereas the release of sTREM‐1 inhibits mbTREM‐1 mediated neutrophil migration and cytokine production and prevents systemic inflammation 128. As sTREM‐1 can be measured in biological fluid only under infection‐induced inflammatory conditions 129, numerous studies have investigated the role of TREM‐1 in the diagnosis and prognosis of sepsis. Serum and urine sTREM‐1 have been suggested to be more sensitive than white blood cell counts, serum CRP and serum procalcitonin (PCT) for the early diagnosis of sepsis induced at different primary infection sites 130, 131. In neonatal sepsis, elevated sTREM‐1 is considered an early marker which can reflect severity and poor prognosis of neonatal sepsis 129. Plasma levels of sTREM‐1 at admission could also be used as a marker to identify patients with a poor prognosis as the sTREM‐1 levels in non‐survivors with severe sepsis who even received early goal‐directed therapy after admission remained high until death 132. In cancer patients with sepsis who might display a more complexed immune profile, the plasma level of sTREM‐1 is still a good predictor of 28‐day mortality 133. When compared with other biomarkers such as PCT and CRP in predicting survival of patients with septic shock, sTREM‐1 was found to be beneficial 134. Other relevant biomarkers related to neutrophils such as neutrophil gelatinase‐associated lipocalin have also been suggested to play roles in the diagnosis and prognosis of sepsis 135, 136 and were reviewed elsewhere 137.

As neutrophils mature in the bone marrow over several stages and healthy individuals do not have immature neutrophils in peripheral blood 138, the potential of simply accounting the percentage and number of immature neutrophils seen in septic patients as a diagnostic biomarker of sepsis has also been studied. It was demonstrated that the number of immature granulocytes count after major burns had the capacity to discriminate between patients with SIRS and sepsis with a sensitivity of 89.2% and a specificity of 76.4% 138. In addition, the immature/total neutrophil ratio was also suggested to enhance the prediction of early onset sepsis in newborns 139. As neutrophils in septic animals and patients displayed abnormal bactericidal activity 16, 140 and a spontaneous migratory phenotype 141, the capacity of neutrophil function together with immature neutrophil count in predicting sepsis has also been evaluated. Studies have shown that the combined use of immature neutrophil count and the neutrophil phagocytic index detected by flow cytometry at day 1 after major burn injury had excellent discriminatory power between septic and non‐septic patients with SIRS with an AUROC of 0.921 16. Moreover, the delta neutrophil index, which is the immature granulocyte fraction and determined by subtracting the fraction of mature polymorphonuclear leucocytes from the sum of myeloperoxidase‐reactive cells, is also an useful marker for early diagnosis and assessment of prognosis in patients with sepsis 142, 143. Taken together, these results suggest that targeting neutrophils may be a promising strategy to diagnose sepsis and/or predict the prognosis of these patients.

## Concluding remarks

Sepsis is a systemic inflammatory syndrome resulting from the immune system response to invading micropathogens and is the leading cause of death among patients in intensive care units. As more than 40 clinical trials which targets on the pro‐inflammatory process during sepsis have failed to improve patient survival, no specific therapy has been developed, with the exception of antibiotic use, fluid resuscitation and supportive systems. With more attention on the role of immunosuppression process in sepsis, therapeutic strategies have gradually focused on immune modulatory treatment to boost antimicrobial immunity. As neutrophils are the key cell subpopulation against invading pathogens, the crucial role of neutrophils in sepsis has been identified as a disturbance in neutrophil recruitment which is correlated with the severity of sepsis. Reprogramming of the migration and function of neutrophils in sepsis has also been studied and was found to improve the survival in animal models. Therefore, further studies into the mechanisms of neutrophil dysregulation and therapeutic targets on neutrophils in sepsis may have potential in sepsis management.

## Funding source

This work was supported by the National Natural Science Foundation of China (No. 81571563, JF Du, and 81500432, XF Shen) and the Fundamental Research Funds for the Central Universities (No. 021414380106, XF Shen).

## Conflict of interest

The authors declare that they have no competing interests.
